# Carbon dioxide sensing in an obligate insect-fungus symbiosis: CO_2_ preferences of leaf-cutting ants to rear their mutualistic fungus

**DOI:** 10.1371/journal.pone.0174597

**Published:** 2017-04-04

**Authors:** Daniela Römer, Martin Bollazzi, Flavio Roces

**Affiliations:** 1 Department of Behavioral Physiology and Sociobiology, Biocenter, University of Würzburg, Am Hubland, Würzburg, Germany; 2 Unidad de Entomología, Departamento de Protección Vegetal, Facultad de Agronomía, Universidad de la República, Montevideo, Uruguay; University of Arizona, UNITED STATES

## Abstract

Defense against biotic or abiotic stresses is one of the benefits of living in symbiosis. Leaf-cutting ants, which live in an obligate mutualism with a fungus, attenuate thermal and desiccation stress of their partner through behavioral responses, by choosing suitable places for fungus-rearing across the soil profile. The underground environment also presents hypoxic (low oxygen) and hypercapnic (high carbon dioxide) conditions, which can negatively influence the symbiont. Here, we investigated whether workers of the leaf-cutting ant *Acromyrmex lundii* use the CO_2_ concentration as an orientation cue when selecting a place to locate their fungus garden, and whether they show preferences for specific CO_2_ concentrations. We also evaluated whether levels preferred by workers for fungus-rearing differ from those selected for themselves. In the laboratory, CO_2_ preferences were assessed in binary choices between chambers with different CO_2_ concentrations, by quantifying number of workers in each chamber and amount of relocated fungus. Leaf-cutting ants used the CO_2_ concentration as a spatial cue when selecting places for fungus-rearing. *A*. *lundii* preferred intermediate CO_2_ levels, between 1 and 3%, as they would encounter at soil depths where their nest chambers are located. In addition, workers avoided both atmospheric and high CO_2_ levels as they would occur outside the nest and at deeper soil layers, respectively. In order to prevent fungus desiccation, however, workers relocated fungus to high CO_2_ levels, which were otherwise avoided. Workers’ CO_2_ preferences for themselves showed no clear-cut pattern. We suggest that workers avoid both atmospheric and high CO_2_ concentrations not because they are detrimental for themselves, but because of their consequences for the symbiotic partner. Whether the preferred CO_2_ concentrations are beneficial for symbiont growth remains to be investigated, as well as whether the observed preferences for fungus-rearing influences the ants’ decisions where to excavate new chambers across the soil profile.

## Introduction

Symbioses, through evolutionary processes, have shaped the biology of many organisms on this planet because they enable the associated organisms for instance to occupy new ecological niches, to gain access to alternative food sources or to attenuate environmental stress. The defense against environmental stressors, be they biotic or abiotic, can be one of the major benefits of a mutual symbiotic association. A classic example of attenuation of biotic stress is the defense of plants by their symbiotic partner against herbivory or competing plants [1–6]. Mutualism also enhances partner fitness by dampening abiotic stress, ranging from solar radiation or nutrient availability to drought or temperature stress [7, 8].

Leaf-cutting ants are a classical example of a successful symbiosis because of their association with a fungus, which rendered them the primary herbivores of the Neotropics [9, 10]. They forage large quantities of live plant material, on which they grow a basidiomycetic fungus as a food source to raise the colony’s brood [11]. Throughout 50 Mio years of evolution of fungus farming [12], the association between the higher Attini leaf-cutting ants and their fungus has become obligate. Yet, biotic and abiotic stressors continuously threaten this successful ant-fungus symbiosis.

Leaf-cutting ants defend against biotic stressors that threaten the fungus garden, like pathogens or parasitic fungi, with an intricate system of pathogen control [13]. It starts outside of the nest with the cleaning of harvested plant material [14], and continues inside the nest with the use of antimicrobial secretions [15] and the removal of pathogenic material from the fungus gardens [16, 17] to special dump sites [18, 19]. Ants also protect their fungal mutualist from the negative effects of unsuitable plants by discontinuing the collection of such material, sometimes for several weeks, via a process involving robust avoidance learning responses [20–25].

Leaf-cutting ants inhabit underground nests consisting of dome-shaped chambers connected to a network of tunnels. In many species, the most superficial fungus chambers are excavated very close to the soil surface [26–28]. In some other species, however, fungus chambers can be found down to a depth of 5–7 meters [18, 29, 30], and their nests can reach huge dimensions with thousands of chambers (*Atta laevigata*; [29]).

The environmental conditions of the soil surrounding the nest influence its climate, and ant workers will encounter gradients across the soil profile, mainly of temperature, moisture, and gases like carbon dioxide [31], putting the fungal symbiont under abiotic stress. As a general pattern, soil moisture increases and soil temperature decreases with depth, and they fluctuate more strongly in the upper soil regions than at deeper layers because of the incoming solar radiation [32]. Therefore, leaf-cutting ant workers should choose places that offer a well suited microclimate when excavating new chambers or deciding where to culture their fungus inside an existing nest. Workers could also avoid desiccation of the fungus and attenuate temperature stress throughout the season by relocating fungus gardens between superficial and deeper soil layers [26, 27, 33], which is likely the reason why empty chambers are found in leaf-cutting ant nests [18, 27–29, 34].

The symbiotic fungus only develops properly at warm temperatures (20–30°C) and high humidity, as it is very prone to desiccation [35, 36]. In order to improve fungal growth, leaf-cutting ants exhibit behavioral responses for the control of the nest microenvironment. In a choice experiment, workers of the grass-cutting ant *Acromyrmex heyeri* preferred to culture their fungus at temperatures between 22–26°C [37], which should ensure proper fungus growth [35, 36]. *Atta sexdens rubropilosa* workers chose places with high humidity values (98%) for fungus culture when given the choice between high and low ones [38]. To prevent humidity losses, leaf-cutting ants also engage in regulatory building responses and close nest openings [39, 40]. When excavating, workers of *Acromyrmex lundii* stop digging at temperatures below 20°C and above 30°C [41], although these temperatures do not correspond to the workers’ physiological thermal limits [42, 43]. It is, however, the temperature range that maximizes fungus growth [35, 36].

The fungus garden appears to be under stress at high levels of CO_2_ as they occur underground, which were shown to negatively influence its respiration rate [44]. A similar effect of CO_2_ on a symbiotic fungus has been found in fungus-rearing termites of the genus *Macrotermes* [45]. Unlike the low levels of CO_2_ in the earth’s atmosphere (currently ~0.04%), levels underground are high and rapidly increase with depth [46, 47], as decaying organic matter, microbial and root respiration generate large CO_2_ amounts, and soil compaction and wetting hinder gas exchanges [48, 49]. The consumption of O_2_ and production of CO_2_ by the underground-nesting ants themselves [50, 51], as well as the CO_2_ produced by the symbiotic fungus, should further increase the hypoxic and hypercapnic conditions underground. Although the underground O_2_ and CO_2_ levels in the nest chambers are in the range observed in the adjacent soil phase [30], they can be influenced to some degree by a wind-induced, passive ventilation mechanism taking advantage of the differences in the elevation of nest openings [30, 52]. Depending on both the depth and the extent of ventilation, CO_2_ levels in leaf-cutting ant nests can vary from 1–2.7% close to the soil surface (~0.5m; unpublished data; [30]) to hypercapnic CO_2_ levels of up to 6% in deeper nest regions [30, 44]. Therefore, leaf-cutting ants should be able to relocate the symbiotic fungus across the soil profile to attenuate the stress of unsuitable CO_2_ concentrations, seeking for proper levels to rear their fungus.

Not only the symbiont is under stress by exposure to increased CO_2_ levels. These can also have long lasting effects on insect physiology, interfering with growth and development, mating behavior, memory retention, and causing water losses [53]. The perception of CO_2_ is very common in insects [53], which possess CO_2_ receptors situated either on the mouthparts or on their antennae [54–56]. Leaf-cutting ants have a special type of chemoreceptor on their antennas, and they perceive not only the relative, but also the absolute CO_2_ concentration of the environment [57, 58], a capability so far unknown for any other insect species.

A number of studies have shown that insects can use CO_2_ as a cue for orientation. Phytophagous and hematophagous insects orient towards CO_2_ to find suitable plant or mammalian hosts for feeding [59–61]. The behavioral responses of ants are far less explored. Workers of the ant *Solenopsis geminata* use CO_2_ as an orientation cue towards buried nestmates [62], and *Cataglyphis* desert ants and leaf-cutting ants can use CO_2_ cues for orientation towards the nest entrance [63, 64].

The special CO_2_ sensilla on the leaf-cutting ants’ antennae would enable workers to continuously monitor CO_2_ levels in their vicinity. It is an open question whether the perception of absolute CO_2_ levels can elicit a behavioral response during in-nest tasks. Given that high CO_2_ concentrations hinder the respiration of the symbiotic fungus [44] and therefore compromise its growth, workers may be able to attenuate hypercapnic stress by relocating the fungus to soil depths with suitable CO_2_ levels. In this study, we investigated whether leaf-cutting ants (*A*. *lundii*) use the CO_2_ levels inside the nest as an orientation cue for the selection of places to relocate their fungus, and quantified CO_2_ choices for fungus cultivation. We also investigated whether workers’ preferences for the fungus differ from those they show for themselves when not engaged in fungus tending. For that, ants were confronted with a binary choice between two interconnected nest chambers offering different CO_2_ concentrations. They encompassed atmospheric values, low levels as those found in superficial soil layers were nests of this species are located, and high levels as found in deeper soil strata where no nest chambers of this superficially nesting species are found. The amount of relocated fungus and the number of workers present in the chambers were quantified as a measure of workers’ CO_2_ preference for fungus rearing, and for themselves, respectively.

## Materials and methods

### Study animals

Colonies of the leaf-cutting ant *A*. *lundii* inhabit shallow subterranean nests, located 30–50 cm underground [65] in heavy clayish soils, where the CO_2_ concentrations range from 1–3% (unpublished measurements). All assays were performed with worker groups collected from laboratory colonies reared in a climatic chamber at 25°C, 50% air humidity and a 12L:12D cycle, and fed *ad libitu*m with blackberry leaves (*Rubus fruticosus*), water and honey water. Colonies were collected near Montevideo, Uruguay, and were brought to the laboratory at the University of Würzburg, Germany. The species *A*. *lundii* is not endangered nor protected. Export permits were issued by the Departamento de Fauna de la Dirección General de Recursos Naturales Renovables, Ministerio de Ganadería, Agricultura y Pesca, Uruguay. The colonies were mature (being at least 4 years old at the time of the experiments) and were kept in a system of closed plastic boxes (19x19x9cm) as artificial fungus chambers, a waste disposal box and a feeding arena, all connected by transparent plastic tubing. The worker groups were collected from the colonies on the day of the assay and not introduced back into the mother colony.

### Experimental setup

In each assay, a group of ants was induced to relocate fungus by exposing it to suboptimal humidity values and offering two potential nest chambers. These chambers presented equal and suitable temperature (~24°C) and humidity values (99.9%), but differed in the CO_2_ concentration.

**Fig 1:**
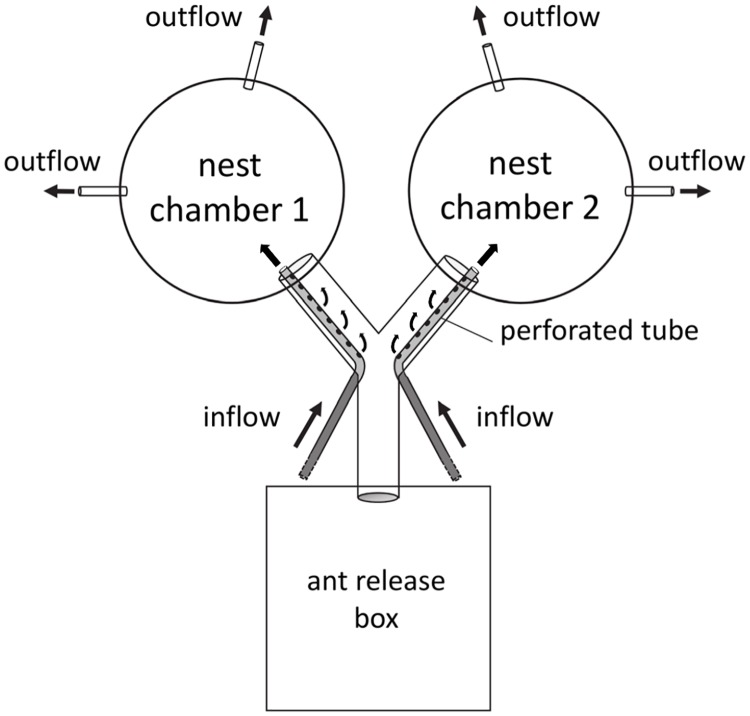
Experimental setup of choice assays. Open box (ant release box) with y-shaped tunnel leading to two nest chambers. Inflow of air with different CO_2_ levels into the chambers took place through small rubber hoses inserted in the bifurcation point of a y-shaped tunnel leading to the nest chambers.

The experimental setup was as follows: A square (9.5x9.5x5.5 cm), open plastic box (ant release box) was connected with a y-shaped tube (y-arm length 6 cm, y-stem length 7 cm, diameter 1.7 cm) to two nest chambers (Fig 1). Each nest chamber consisted of a plastic ring (diameter 10 cm, height 3 cm), with a glass bottom (10x10 cm), and a lid made out of clear plastic. A moistened piece of filter paper (diameter 10 cm) was placed on the bottom of each chamber to increase the air humidity to values well suited for fungus rearing (99.9%, n = 8; [38]).

To establish the different atmospheres in the chambers, air from two independent sources having different CO_2_ concentrations was pumped into the setup at a flow rate of 50 ml*min^-1^, starting at the bifurcation point of the y-tube (Fig 1). Here, two small rubber hoses (diameter 0.3 cm) ran along the inner walls of the y-tube and ended inside each chamber. To generate different CO_2_ levels already at the y-bifurcation, the hoses were perforated with small holes, allowing part of the injected air to leak out into the tube so it could be used as an orientation cue. For atmospheric levels, the standard laboratory compressed air was used as source. The different elevated CO_2_ concentrations were generated by mixing compressed air with pure CO_2_ using a gas-mixing device (Mass Flow Controller MFC-4, Sable Systems International, USA). The air in the chambers was then pumped out (miniature vane pump, 135 FZ, Schwarzer Precision, Germany) at an equal flow rate of 50 ml*min^-1^ through two rubber hoses (diameter 0.3 cm) inserted in the opposite chamber wall of the chamber entrance. It is important to indicate that due to some slight mixing of the two independent airstreams leading to the chambers, the mean CO_2_ level in the chambers intended to have atmospheric values was slightly higher, reaching values from 0.06% to 0.29% in the different experiments, still far below the lowest CO_2_ concentration (1%) used as the alternative choice.

### Experimental series

Three different series with a total of eleven experiments were performed to investigate both the use of CO_2_ as orientation cue for fungus placement and the range of preferred CO_2_ concentrations. The CO_2_ levels offered as choice included atmospheric values, low levels as those measured in field nests of *A*. *lundii* (1–3%, unpublished results), and high levels (4%) as measured in deeper nests of the genus *Atta* [30].

Series 1 –Choice between atmospheric and elevated CO_2_ concentrations: experiments: atmospheric vs 1%, atmospheric vs 2%, atmospheric vs 3%, and atmospheric vs 4%.

Series 2 –Choice between intermediate CO_2_ concentrations, and high CO_2_ concentrations: experiments: 1% vs 2%, 1% vs 3% and 1% vs 4%.

Series 3 –Choice between high CO_2_ concentrations: experiments: 2% vs 3%, 2% vs 4%, 3% vs 4%, 4% vs 4% (control for side bias).

### Experimental procedure

The assays were performed as follows. One hundred media-sized workers were collected in equal numbers out of the feeding box and a randomly chosen fungus garden box of one of the four colonies (an overview of number of assays per colony is presented in Table A, S1 File). In addition, 1 g fungus was removed from the fungus garden, and all ants and brood in it were carefully removed. Once the CO_2_ concentrations for a given assay were established in the two nest chambers (measurements were done with a CO_2_ sensor: Gasmitter, Sensor Devices, Germany; range 0–10%, resolution: 0.01%), the collected 100 workers were placed into the release box. Workers could freely enter the two nest chambers with the different CO_2_ concentrations and explore them. After 1h, the number of ants present in each chamber was counted. Immediately thereafter, the collected fungus was divided into small pieces (mean weight 46.22 mg, SD = 4.03, n = 20) and placed into the open ant release box. Here the fungus was exposed to room conditions with an air humidity between 30–45%, so that ants were expected to relocate the fungus pieces to a more suitable site because of desiccation risks. Workers had then 3 hours to relocate the fungus inside the chambers according to their CO_2_ preferences. Thereafter, the number of workers present in each chamber was counted again and the relocated fungus was collected and dried for 24 h at 50°C. Because of the initial familiarization period of 1 hour before placing the fungus into the ant release box, where workers were observed to calmly explore the setup, we are confident that the observed fungus relocations and CO_2_ choices are representative responses to avoid fungus desiccation as they occur under natural conditions. The mass of the dry fungus was weighed to the nearest 0.1 mg. To avoid side biases, the sides with the differing CO_2_ concentrations were alternated between replicates.

Depending on their normal distribution, datasets were compared using either the Wilcoxon matched pair test or the paired t-test. However, all data was presented as box-plots with medians for the sake of homogeneity, even if particular datasets were normally distributed.

## Results

After their release in the open plastic box, workers immediately started exploring the y-shaped tubing and the two nest chambers. Workers moved back and forth between them and the ant release box and did not remain or aggregate inside the more humid chambers. At any given time, only some workers were present in the two nest chambers, while others were moving in the y-shaped tubing or present in the release box.

When the fungus was placed in the release box, several workers from the box and others coming from the chambers were observed to explore and aggregate near the fungus. Fungus relocation did not take place immediately and usually occurred after 30 to 60 minutes (S1 Video in the supporting information shows the transport of a piece of fungus into a nest chamber).

### Choice between atmospheric and elevated CO_2_ concentrations

**Fig 2:**
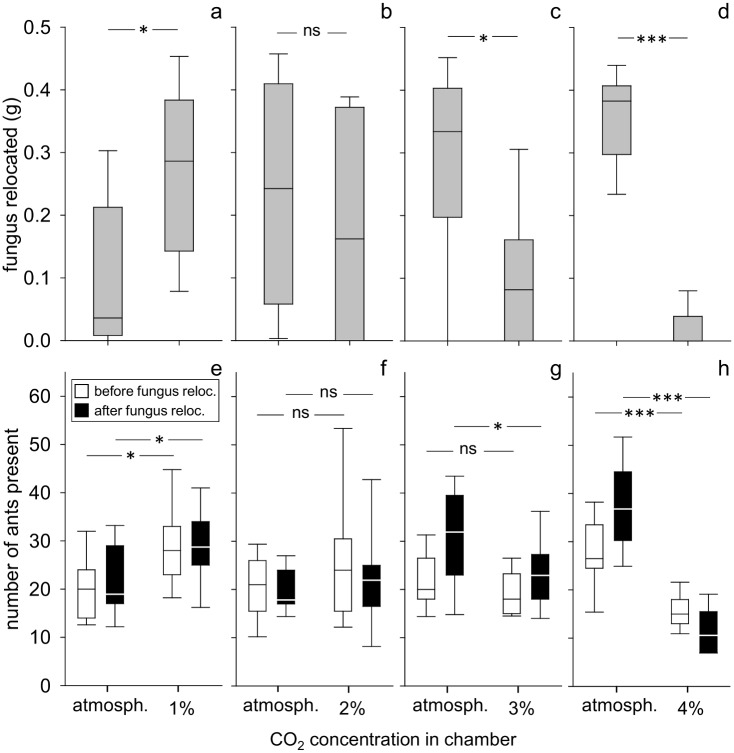
Choice between atmospheric and elevated CO_2_ concentrations. a-d: amount of relocated fungus in the chambers, e-h: number of ants present in chambers; a and e—atmospheric vs 1%, n = 15, b and f—atmospheric vs 2%, n = 21, c and g—atmospheric vs 3%, n = 18, d and h—atmospheric vs 4%, n = 18; box: 25–75% percentiles, line: median, whiskers: min-max values; ns = not significant, *p ≤ 0.05, **p<0.01, ***p<0.001

When presented with a choice between atmospheric levels and 1% CO_2_ for fungus relocation, workers of *A*. *lundii* deposited more fungus in the chamber with 1%. When confronted with a level of 2%, there was no clear choice for either 2% or atmospheric values. Levels of 3% and 4% were avoided (Fig 2a–2d; Wilcoxon matched pair test, atmosph. vs 1%: n = 15, p = 0.015; atmosph. vs 2%: n = 21, p = 0,23; atmosph. vs 3%: n = 18, p = 0.02; atmosph. vs 4%: n = 18, p<0.001; statistical details are provided in Table B, S1 File).

When given a choice between atmospheric values and 1% CO_2_, more workers were present in the chamber with 1% CO_2_. Yet workers were evenly distributed between the two chambers when the offered alternative was 2% CO_2_. With 3% CO_2_ as alternative, workers were also evenly distributed between both chambers before the fungus was offered, but were present in significantly higher numbers in the chamber with atmospheric levels at the end of the assays. With 4% CO_2_ as alternative, more workers could be found in the chamber with atmospheric levels already before the fungus was offered (Fig 2e–2h; atmosph. vs 1%: paired t-test, n = 15, before: p = 0,016, after: p = 0.001; atmosph. vs 2%: n = 21, before: paired t-test, p = 0.97, after: Wilcoxon matched pair test, p = 0.63; atmosph. vs 3%: paired t-test, n = 18, before: p = 0.27, after: p = 0.042; atmosph. vs 4%: Wilcoxon matched pair test, n = 18, before: p = 0.0004, after: p<0.001). Across the experiments, the number of ants present in each chamber before the fungus was offered corresponded well with the distribution pattern after fungus relocation, with the exception of the experiment ‘atmospheric values vs 3%’. At the end of the experiments, the pattern of worker distribution always corresponded with the distribution pattern of the fungus, i.e., when the fungus was equally distributed between the two chambers, ants were also evenly distributed; when one chamber was preferred for fungus relocation, more ants were present in that chamber (Fig 2a–2d and 2e–2h, black box-plots).

### Choice between intermediate and high CO_2_ concentrations

**Fig 3:**
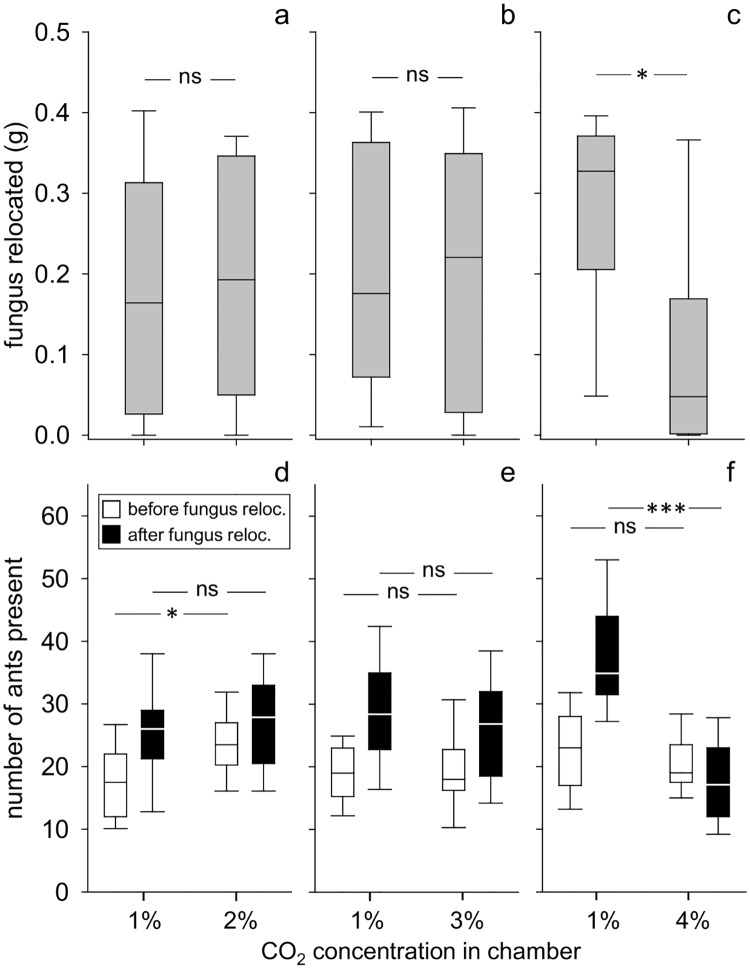
Choice between 1% CO_2_, as it occurs at superficial soil layers, and higher concentrations. a-c: amount of relocated fungus in chambers, d-f: number of ants present in chambers; a and d– 1% vs 2%, n = 20, b and e– 1% vs 3%, n = 20, c and f– 1% vs 4%, n = 21; box: 25–75% percentiles, line: median, whiskers: min-max values; ns = not significant, *p ≤ 0.05, **p<0.01, ***p<0.001

In the previous series, a value of 1% CO_2_ was preferred to atmospheric levels. In the present series, when ants had the choice between 1% CO_2_ in one chamber and either 2% or 3% in the other, there were no differences in fungal deposition between either 1% vs 2%, or 1% vs 3%. However, workers avoided 4% for fungus relocation and chose the alternative chamber with 1% CO_2_ (Fig 3a–3c; Wilcoxon matched pair test, 1% vs 2%: n = 20, p = 0.68; 1% vs 3%: n = 20, p = 1.0; 1% vs 4%: n = 21, p = 0.012; statistical details in Table C, S1 File).

In the experiments where both chambers offered a CO_2_ environment as encountered at superficial soil layers (1% vs 2%), ants initially chose the higher CO_2_ level of 2%, i.e., before the fungus was offered. In the other two choice experiments (1% vs 3% and 1% vs 4%), ants did not show an initial preference for themselves for one of the two CO_2_ levels. After fungus relocation, the ant distribution always followed the pattern of the fungus distribution (Fig 3d–3f; paired t-test, 1% vs 2%: n = 20, before: p = 0.006, after: p = 0.58; 1% vs 3%: n = 20, before: p = 0.93, after: p = 0.31; 1% vs 4%: n = 21, before: p = 0.26, after: p<0.001).

### Choice between different high CO_2_ concentrations

**Fig 4:**
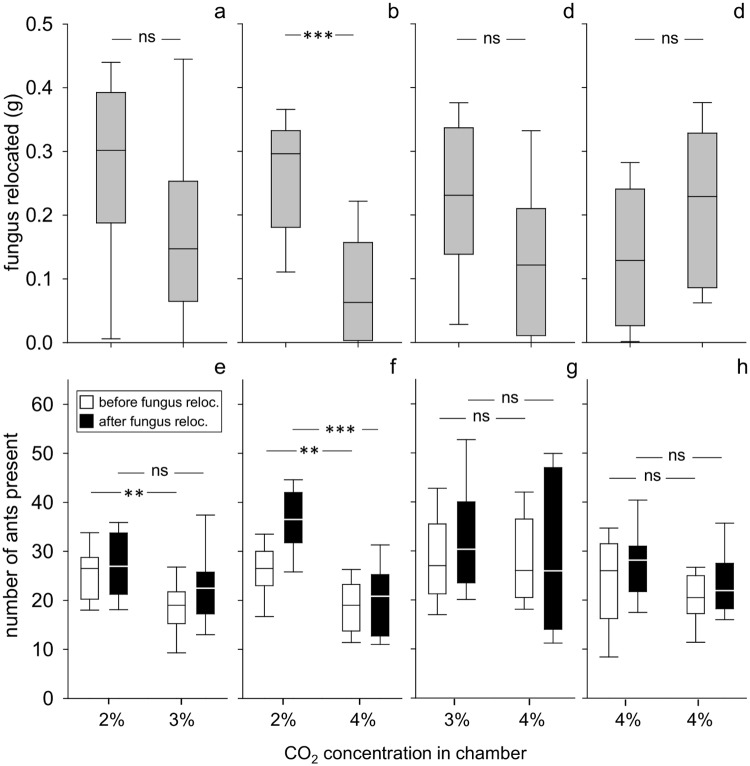
Choice between high CO_2_ concentrations as they occur at deeper soil layers. a-d: amount of relocated fungus in chambers, e-h: number of ants present in chambers; a and e– 2% vs 3%, n = 20, b and f– 2% vs 4%, n = 26, c and g– 3% vs 4%, n = 20, d and h– 4% vs 4%, n = 12; box: 25–75% percentiles, line: median, whiskers: min-max values; ns = not significant, *p ≤ 0.05, **p<0.01, ***p<0.001

Workers evenly distributed the fungus between chambers with 2% and 3% CO_2_, and also between 3% and 4% CO_2_. However, they avoided 4% for fungus relocation when the alternative nest site offered a level of 2%. Fungus and workers were also evenly distributed in the control experiment with high CO_2_ values (4%) in both chambers, indicating no side bias (Fig 4a–4d; 2% vs 3%: paired t-test, n = 20, p = 0.153; 2% vs 4%: Wilcoxon matched pair test, n = 26, p<0.001; 3% vs 4%: Wilcoxon matched pair test, n = 20, p = 0.1; 4% vs 4%: paired t-test, n = 12, p = 0.22; statistical details in Table D, S1 File).

In three of the four experiments of this series (2% vs 4%, 3% vs 4% and 4% vs 4%), the pattern of ant distribution before and after fungus relocation did not differ. Workers chose 2% CO_2_ when offered either 2% vs 3% or 2% vs 4%, but distributed evenly when higher CO_2_ concentrations, as they occur at deeper soil layers, were offered (3% vs 4% and 4% vs 4%). In all four experiments, the distribution pattern of workers after fungus relocation corresponded to that of the fungus (Fig 4e–4h; paired t-test, 2% vs 3%: n = 20, before: p = 0.005, after: p = 0.14; 2% vs 4%: n = 26, before: p = 0.001, after: p<0.001; 3% vs 4%: n = 20, before: p = 0.7, after: p = 0.38; 4% vs 4% (control): n = 12, before: p = 0.31, after: p = 0.18).

It is important to indicate that in some experiments, even though the mean proportion of the relocated fungus did not differ between the two alternatives, the proportion observed in each single assay deviated from a 1:1 ratio. In some assays, a bias in favor of one or the other chamber was observed. We therefore evaluated, across all assays, how often a given proportion of relocated fungus was observed in one of the offered alternatives. Results showed that in experiments with no clear choice for one of the CO_2_ levels (i.e., atmosph. vs 2%, 1% vs 2%, 1% vs 3%, 2% vs 3%, 3% vs 4% and 4% vs 4%), the fungus was not always equally distributed in each single assay, but mainly relocated into one of the chambers (Supporting information S1 File, Fig A, subfigure b, Fig B, subfigure a and b, Fig C, subfigures a-c). It appeared that workers continued piling fungus into the chamber where the first pieces had been relocated to, resulting in an uneven fungus distribution. The preferences displayed in single assays later cancelled out as means were calculated. In experiments with a significant average preference for a given CO_2_ value, however, the majority of single assays also displayed preferences for that value (Supporting information S1 File, Fig A, subfigure a and d, Fig B, subfigure c and Fig C, subfigure b).

## Discussion

Our results demonstrated that leaf-cutting ants show preferences for a specific range of CO_2_ concentrations when relocating their symbiotic fungus. They preferred intermediate CO_2_ levels (1–3%) and avoided both atmospheric and high CO_2_ levels (4%). Such preferences did not necessarily correspond to the preferences ants showed for themselves. Instead, values were chosen for fungus rearing, probably to attenuate the symbiont’s abiotic stress. However, workers traded off their CO_2_ preferences and selected high CO_2_ levels for fungus maintenance, levels that were otherwise avoided, in order to prevent another abiotic stressor such as low relative humidity.

### Preference and avoidance of CO_2_ concentrations for fungus rearing

*A*. *lundii* workers avoided high CO_2_ concentrations for fungus rearing. Measurements in leaf-cutting ants and fungus-farming termites showed that high CO_2_ concentrations hinder the respiration rate of the symbiont [44, 45]. Since workers of another *Acromyrmex* species, *A*. *ambiguus*, also avoided high CO_2_ values for fungus rearing [66], this behavior appears to be a general, robust response of *Acromyrmex* leaf-cutting ants to control the environment for their fungus culture. Although their responses were not as strong as for high CO_2_ values, workers also avoided the relocation of fungus to atmospheric CO_2_ levels, unless confronted with very high CO_2_ concentrations as alternative. It is unknown whether atmospheric levels are directly detrimental to fungus growth, yet this appears unlikely. We speculate that atmospheric levels are avoided because they are only found in the outside environment, usually in association with more variable temperature and humidity values, which may reach suboptimal levels.

In our experiments, intermediate CO_2_ levels (1–3%) were chosen for fungus culture, in the range measured in the superficial soil layers where colonies of this species excavate their nest chambers (unpublished data). This indicates that *A*. *lundii* prefers values usually experienced inside their nests. While information about *in vitro* growth rates of the symbiotic fungus under different CO_2_ concentrations is lacking, we speculate that workers chose certain CO_2_ levels to improve fungus growth, as temperature and humidity values chosen in laboratory experiments [37, 38] correspond to values that maximize fungus growth *in vitro* [35, 36]. As studies with other, non-symbiotic fungi have shown, fungus growth appears to be first facilitated as the CO_2_ concentrations increase, and then hindered at higher concentrations [67, 68]. The chosen CO_2_ levels might also change the pH of the nest soil the fungus is resting on towards more favorable levels, promoting better fungal growth. Levels of pH between 4.5 and 5 have been shown to increase fungal gongylidia growth *in vitro*, the food of the leaf-cutting ant brood [35].

It is important to indicate that throughout our manuscript, we have used the word ‘preference’ to describe the selection of a given CO_2_ concentration, as previously used in other publications dealing for instance with temperature and humidity selection [38, 69, 70]. Whether absolute preferences for specific levels or ranges exist remains elusive. The observed selection of one of the two alternatives in our experiments, or even the selection along a continuous gradient of a given variable (i.e., [37]) could be rather based on avoidance of the less suitable alternative in a choice situation or on avoidance of unsuitable low and high values. Whatever the underlying mechanisms, the preference for intermediate CO_2_ levels indicates that leaf-cutting ants can detect absolute CO_2_ concentrations, as previously demonstrated [57].

### Acceptance of high CO_2_ levels

Our control experiment with the high CO_2_ concentration of 4% in both chambers showed that workers accepted high CO_2_ levels for themselves and for fungus rearing, in order to avoid desiccation, levels that were otherwise avoided and are known to negatively influence fungus respiration [44]. It is tempting to hypothesize that workers selected a high CO_2_ concentration as a cue that indirectly indicates stable nest conditions over time and also a well isolated nest space. As a result, workers may indirectly avoid desiccation risks via ventilatory airflows through the nest, even though ants are known to counteract nest humidity losses by building behavior [39].

Underground environmental variables such as temperature, soil moisture/relative humidity and CO_2_, fluctuate differently with latitude, soil depth, weather and time of year [32]. For example, levels of soil moisture and CO_2_ increase with depth. Therefore, humidity levels well-suited for fungus growth at one site, i.e., at deep soil layers, do not necessarily imply proper ranges of other abiotic factors at that site. Leaf-cutting ants should have adapted their fungus-tending behavior to cope with trade-offs between their environmental preferences. When excavating a new fungus chamber, or relocating fungus between already existing chambers, leaf-cutting ants should choose a site offering the best possible environment for fungus growth. As a result of such trade-offs, ants may sometimes select for instance deep soil layers for fungus culturing to avoid the dry conditions that occur at superficial soil layers, at the expense of experiencing high CO_2_ levels that negatively influence fungus growth.

Besides the behavioral adaptation of relocating the fungus to avoid unfavorable environmental conditions, physiological adaptations to unfavorable underground conditions may also exist, as for instance the development of higher tolerance to specific environmental variables. To date, no physiological adaptation of leaf-cutting ant workers to tolerate high CO_2_ concentrations are known. Mangrove ants, which face similar high CO_2_ levels as *Atta* leaf-cutting ants due to inundation of their nests [71], can switch to anaerobic respiration when CO_2_ levels increase [72]. Regarding other environmental stressors, both behavioral and physiological adaptations were described for leaf-cutting ant workers and fungus, respectively. Workers of a number of *Acromyrmex* species accumulate dry plant material and soil to form a thatched mound above the fungus chambers, which might help to prevent humidity loss and lessen environmental fluctuations, allowing for fungus culture close to the soil surface [41, 73–75]. The leaf-cutting ant *Atta texana*, which relocates their fungus gardens from cold superficial soil layers into a central chamber deep underground in winter, has developed a more cold-tolerant fungal strain in colonies at its northernmost distribution range [76]. It is tempting to speculate that at least some of the fungal strains of leaf-cutting ant species inhabiting deep nests may have adapted to better tolerate higher CO_2_ levels.

While leaf-cutting ants use the CO_2_ concentration as an orientation cue to select a place for their symbiotic fungus, it seems that other mechanisms can also influence workers’ decision. The side biases in the distribution of relocated fungus observed in some single assays, as described above, indicate that the first relocated fungus piece acted as a cue and influenced the placement of the subsequent ones, likely as a stigmergic response [77]. Workers could perceive the fungus by chemical cues, like hydrocarbons emanating from the fungus [78], or follow trail pheromones laid by initial workers on their way to the chamber. It is unlikely that the CO_2_ produced by the fungus also acts as an orientation cue, since the CO_2_ levels in the fungus chambers and the surrounding soil do not differ [30], likely because the soil is not only a source but also a massive CO_2_ sink.

### CO_2_ preferences of workers for themselves

It could be a priori argued that ants did not show specific CO_2_ preferences to protect their symbiont against environmental stress, but rather relocated the fungus following the CO_2_ preferences for themselves. Co-evolution could have shaped the ants’ environmental preferences to match preferences for fungus growth, as culturing fungus under unsuitable conditions would also be detrimental for colony fitness. Alternatively, studies have shown that ants use CO_2_ as an orientation cue to find their nest [63, 64]. The selected CO_2_ levels in our experiments might have been used as a nest cue for unladen or fungus-carrying workers, thus leading to the accumulation of fungus in the chamber with CO_2_ levels expected to occur inside the nest.

If CO_2_ preferences for fungus relocation were solely based on worker preferences for themselves, we would expect a match between the workers’ CO_2_ choices before fungus was given and the later choices for fungus relocation. However, this was only the case in some of the experiments. In others, CO_2_ preferences for the ants before and after fungus relocation were different. Therefore, there is no clear evidence that the CO_2_ preferences for fungus rearing simply correspond to the workers’ preferences for themselves. Interestingly, worker distribution after fungus relocation into the chambers always matched the observed fungus distribution. As recently demonstrated [79], the symbiotic fungus strongly attracts leaf-cutting ant workers, which may use its odor as an orientation cue [66].

It is an open question whether workers show specific CO_2_ preferences for the developing brood, which is raised embedded in the fungus gardens, and whether these choices do coincide with the choices for fungus rearing. Workers do show preferences for brood rearing temperatures [37] that coincide with the temperature range suitable for fungus rearing. Analogous to fungus, brood is also susceptible to desiccation because of its soft integument [69, 80], and leaf-cutting ant workers relocate brood from low to high air humidity (personal observation). As high CO_2_ concentrations are known to have detrimental effects on insects and their brood, especially on their growth and development [53], choosing a proper CO_2_ environment to attenuate abiotic stress of the developing brood would also ensure colony survival.

### Influence of underground CO_2_ levels on excavation and nest growth

Relocation of the fungus, and likely of brood, can be seen as short-term response to counteract unfavorable CO_2_ values. The development of high tolerance to adverse conditions, as described above, can be instead regarded as a long-term response. Excavating nest chambers in the soil where favorable levels of CO_2_ or other variables for fungus rearing are encountered would lead to a well suited nest environment in the long term. The preferred CO_2_ levels in the nest soil could act as an environmental template during nest building, concentrating the excavation activity, and the emergence of nest chambers, at certain soil layers. Interestingly, a nest-excavation study with the Florida harvester ant *Pogonomyrmex badius* showed no influence of CO_2_ concentrations on the spatial arrangement of the nest chambers; even inversed CO_2_ gradients across the soil profile did not change the nest shape [81]. However, nests of this species occur in well ventilated sandy soils with very low underground CO_2_ levels (even at depth of 1.5 and 1.8 m the measured CO_2_ concentrations underground were only 0.6 and 0.7% CO_2_, respectively), and colonies do not live in symbiosis with a fungal cultivar that is hindered at certain CO_2_ values. For fungus-growing ants, using CO_2_ as an orientation cue while excavating and selecting levels well suited for fungus growth could lead to a long-term response for the attenuation of CO_2_ stress on the symbiont. Other abiotic factors have been shown to influence digging behavior of leaf-cutting ants. Workers of *A*. *lundii* excavated more soil at temperatures between 20–30°C, with a peak performance at 25°C [41], i.e., at the most suitable temperature for fungus growth [35, 36]. The Chaco leaf-cutting ant *Atta vollenweideri* preferred to excavate in moist soils and avoided dry ones [82], which should lead to high humidity values in the nest air. So far, there is no information about the influence of CO_2_ levels on digging behavior in leaf-cutting ants.

Our study demonstrated that leaf-cutting ant workers can use the CO_2_ concentration of their nest environment as a spatial cue for the selection of a place for fungus rearing. The relocation behavior can be seen as a short-term response to attenuate an abiotic stressor to the symbiotic partner. Long-term responses may include the tolerance of suboptimal CO_2_ conditions for fungus rearing by the workers in favor of the control of a more dangerous abiotic stressor like desiccation, and likely the excavation of nest space at appropriate CO_2_ levels, a strategy that awaits experimental exploration.

## Supporting information

S1 Video Worker of *Acromyrmex lundii* relocating fungus. (AVI)

S1 File Supplementary figures and statistical tables. (PDF)

S2 File Complete raw data. (XLSX)
