# Supplementary material for: Carbon dioxide sensing in an obligate insect-fungus symbiosis: CO2 preferences of leaf-cutting ants to rear their mutualistic fungus
Source: PLoS One. 2017 Apr 4;12(4):e0174597. doi: 10.1371/journal.pone.0174597 (PMC5380341; doi:10.1371/journal.pone.0174597)
Supplement: S1 File — (PDF) [file pone.0174597.s002.pdf]

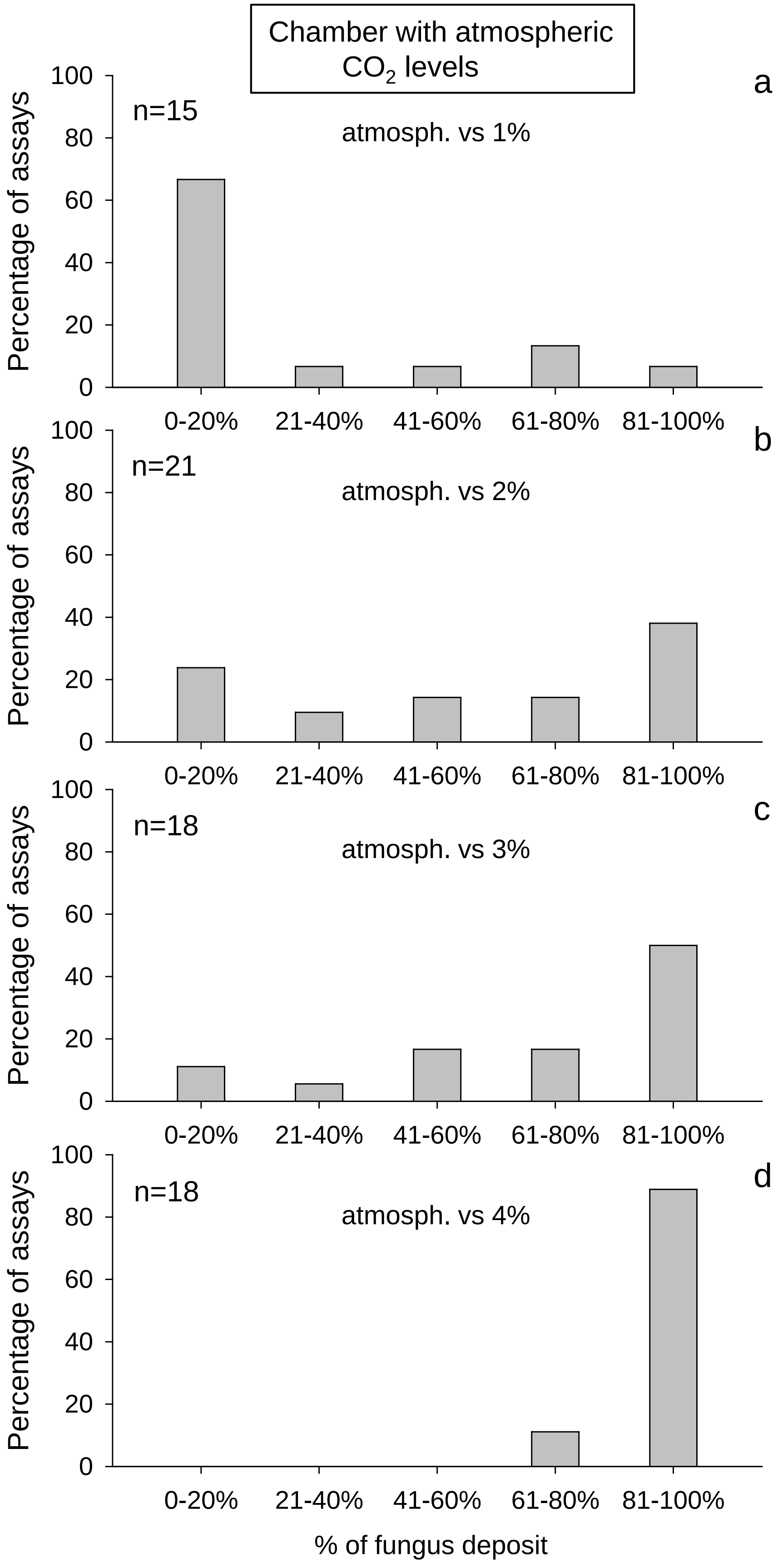

Fig A. Fungus deposit in atmospheric chamber. Percentage of choice assays in which the fungus deposit in the chamber with atmospheric CO_2 levels corresponded to one of the intervals indicated on the x-axis.

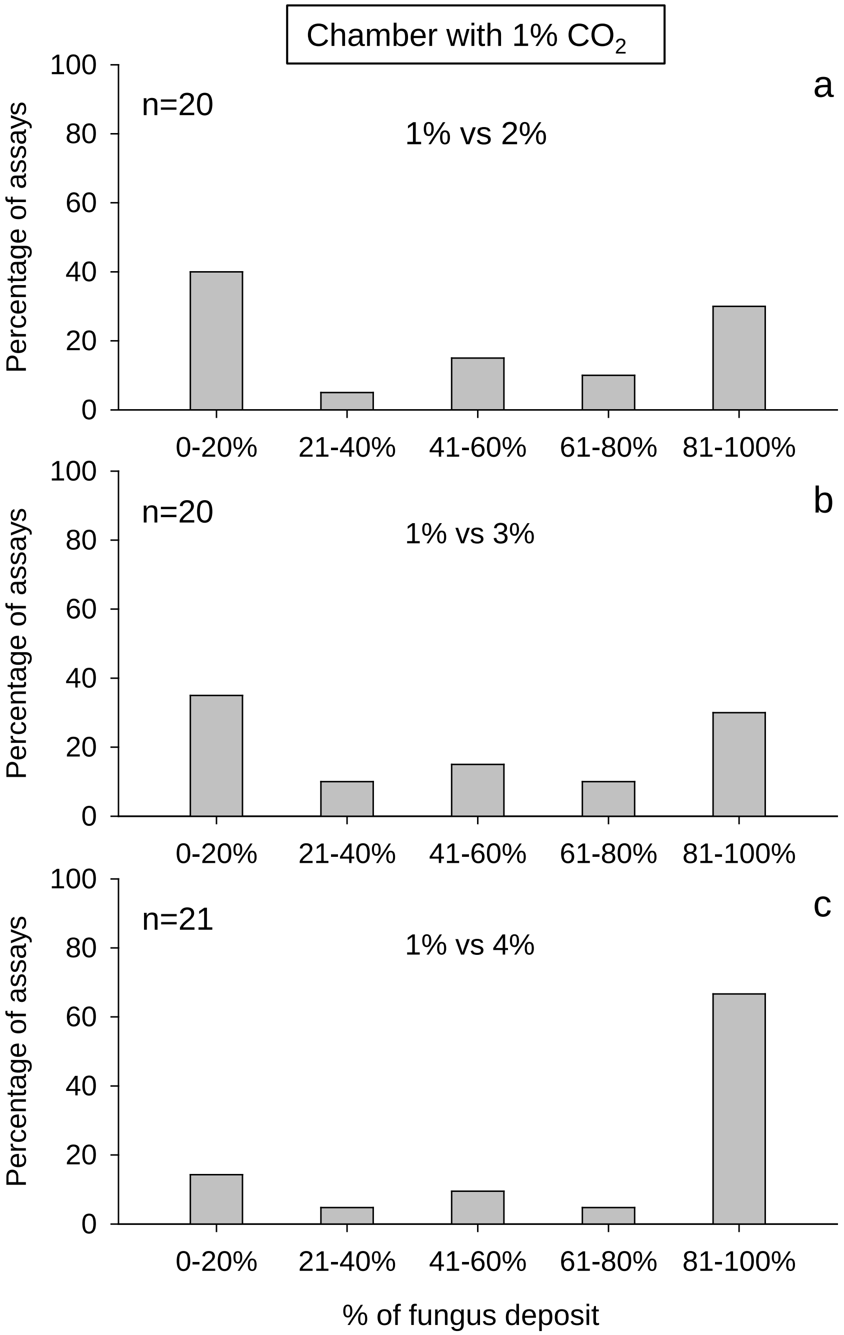

Fig B. Fungus deposit in chamber with 1% CO_2. Percentage of choice assays in which the fungus deposit in the chamber with 1% CO_2 levels corresponded to one of the intervals indicated on the x-axis.

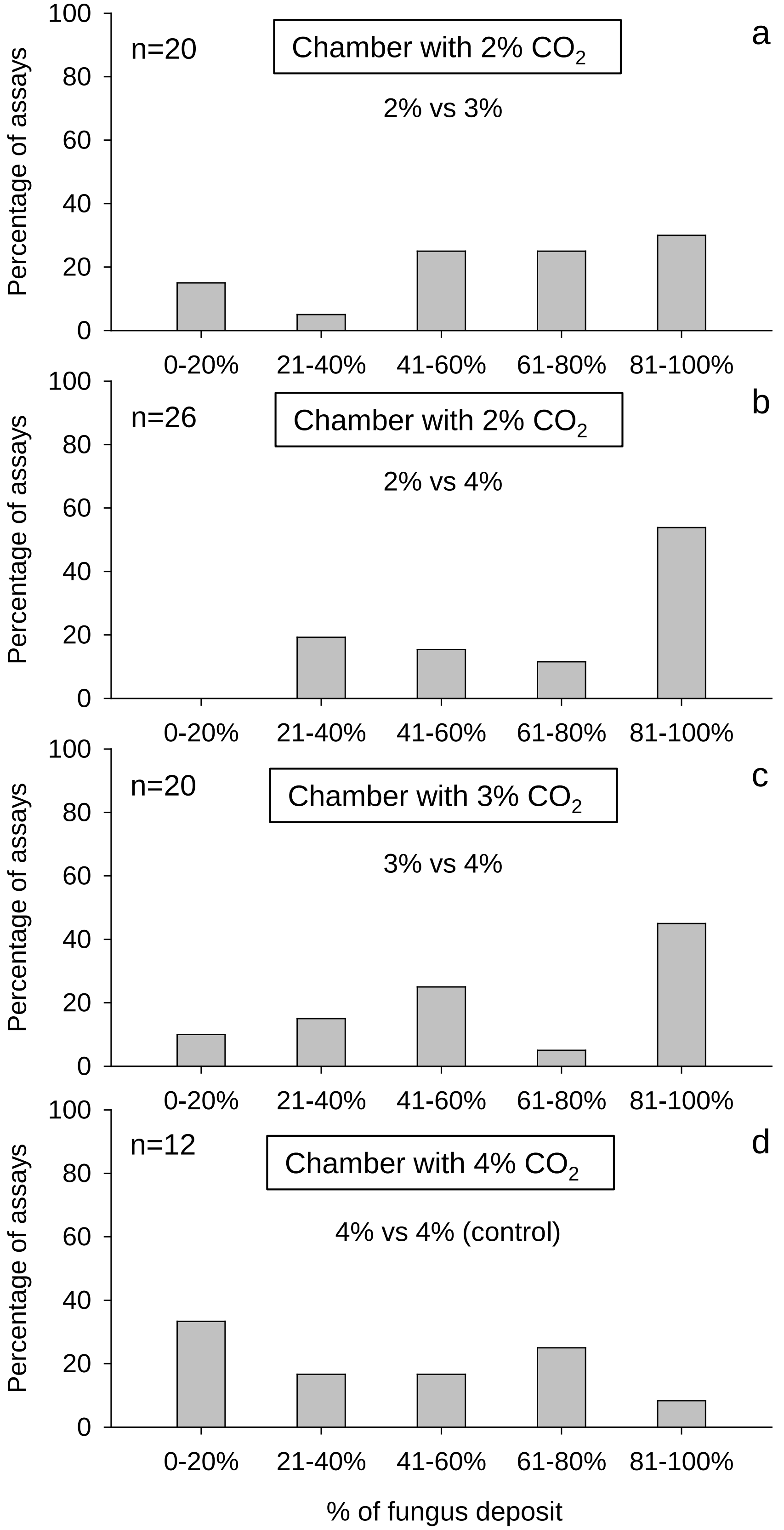

Fig C. Fungus deposit in chamber with lower CO_2 level. Percentage of choice assays in which the fungus deposit in the chamber with the lower CO_2 level corresponded to one of the intervals indicated on the x-axis.

Table A. Overview of number of assays performed per colony

Experiment	Colony 1	Colony 3	Colony 4	Colony 6	n total
atmosph. vs 1%	4	4	3	4	15
atmosph. vs 2%	7	6	4	4	21
atmosph. vs 3%	4	4	6	4	18
atmosph. vs 4%	6	3	4	5	18
1% vs 2%	5	5	5	5	20
1% vs 3%	4	7	4	5	20
1% vs 4%	6	4	6	5	21
2% vs 3%	5	5	7	3	20
2% vs 4%	3	6	8	9	26
3% vs 4%	5	5	5	5	20
4% vs 4%	2	3	4	3	12

Table B. Details statistical analysis series 1, comparing the preferences for either atmospheric or elevated CO_2 levels

atmosph. vs 1%	Test	n			p	
fungus	Wilcoxon matched pair	15	T=17	Z=2.442	p=0.0145	*
ants 1h	paired t-test	15	t=2.726	df=14	p=0.0164	*
ants 4h	paired t-test	15	t=2.981	df=14	p=0.001	***
atmosph. vs 2%	Test	n				
fungus	Wilcoxon matched pair	21	T=81	Z=1.199	p=0.2304	ns
ants 1h	paired t-test	21	t=0.0434	df=20	p=0.97	ns
ants 4h	Wilcoxon matched pair	21	T=83	Z=0.483	p=0.629	ns
atmosph. vs 3%	Test	n				
fungus	Wilcoxon matched pair	18	T=32	Z=2.33	p=0.02	*
ants 1h	paired t-test	18	t=1.13	df=17	p=0.27	ns
ants 4h	paired t-test	18	t=2.19	df=17	p=0.042	*
atmosph. vs 4%	Test	n			p	
fungus	Wilcoxon matched pair	18	T=0.00	Z=3.72	p=0.000196	***
ants 1h	Wilcoxon matched pair	18	T=4.00	Z=3.55	p=0.000386	***
ants 4h	Wilcoxon matched pair	18	T=0.00	Z=3.72	p=0.000196	***

Table C. Details statistical analysis series 2, comparing the preferences for either intermediate CO_2 levels, as they occur at superficial soil layers, or higher concentrations.

1% vs 2%	Test	n			p	
fungus	Wilcoxon matched pair	20	T=94	Z=0.41	p=0.68	ns
ants 1h	t-test	20	t=3.12	df=19	p=0.0056	**
ants 4h	t-test	20	t=0.56	df=19	p=0.58	ns
1% vs 3%	Test	n				
fungus	Wilcoxon matched pair	20	T=105	Z=0.0	p=1.00	ns
ants 1h	paired t-test	20	t=0.091	df=19	p=0.93	ns
ants 4h	paired t-test	20	t=-1.04	df=19	p=0.31	ns
1% vs 4%	Test	n				
fungus	Wilcoxon matched pair	21	T=43	Z=2.52	p=0.012	*
ants 1h	paired t-test	21	t=-1.17	df=20	p=0.256	ns
ants 4h	paired t-test	21	t=-6.66	df=20	p=0.000002	***

Table D. Details statistical analysis series 3, comparing the preferences for high CO_2 levels as they occur at deeper soil layers.

2% vs 3%	Test	n			p	
fungus	paired t-test	20	t=-1.49	df=19	p=0.153	ns
ants 1h	paired t-test	20	t=-3.14	df=19	p=0.0053	**
ants 4h	paired t-test	20	t=-1.53	df=19	p=0.142	ns
2% vs 4%	Test	n				
fungus	Wilcoxon matched pair	26	T=41	Z=3.42	p=0.000635	***
ants 1h	paired t-test	26	t=-3.62	df=25	p=0.0013	**
ants 4h	paired t-test	26	t=-6.70	df=25	p=0.000001	***
3% vs 4%	Test	n				
fungus	Wilcoxon matched pair	20	T=61	Z=1.64	p=0.10	ns
ants 1h	paired t-test	20	t=-0.39	df=19	p=0.7	ns
ants 4h	paired t-test	20	t=-0.89	df=19	p=0.38	ns
4% vs 4%	Test	n				
fungus	paired t-test	12	t=1.31	df=11	p=0.22	ns
ants 1h	paired t-test	12	t=-1.07	df=11	p=0.31	ns
ants 4h	paired t-test	12	t=-1.43	df=11	p=0.18	ns

Video S1. Worker of *Acromyrmex lundii* relocating a piece of fungus. Relocation into a chamber with 1% CO_2 level, in a choice assay.
